# Whether Green Finance Improves Green Innovation of Listed Companies—Evidence from China

**DOI:** 10.3390/ijerph191710882

**Published:** 2022-08-31

**Authors:** Zhao Dong, Haodong Xu, Zhifeng Zhang, Yipin Lyu, Yuqi Lu, Hongyan Duan

**Affiliations:** 1 Institute of Standardization, Qingdao University, Qingdao 266071, China; 2 School of Economics, Qingdao University, Qingdao 266071, China; 3 College of Engineering and Applied Sciences, The State University of New York at Stony Brook, 100 Nicolls Road, Stony Brook, NY 11794, USA; 4 School of Marxism, East China University of Political Science and Law, Shanghai 201620, China; 5 Department of Economics, The University of Sheffield, Sheffield S10 2TN, UK

**Keywords:** green finance reform and innovation, green innovation level, debt financing cost, long-term debt ratio, difference-in-difference model

## Abstract

Facing the intensification of global carbon emissions and the increasingly severe pressure of environmental pollution, listed companies urgently need to promote green innovation, achieve green transformation, and alleviate environmental problems. Green finance policy has played a significant role as a financial strategy for environmental governance in affecting green innovation level over the years. In this context, taking the green finance reform and innovation pilot zone (GFRIPZ) implemented in 2017 in China as a quasi-natural experiment, this paper analyzes the impact of green finance policy on green innovation level of listed companies by the difference-in-difference model. Based on the data of Chinese A-share listed companies from 2008 to 2020, the results of empirical analysis show that green finance significantly promotes green innovation of listed companies. The effect is profound on green utility model patents, but less pronounced on green invention patents. Among all these pilot zones, the policy effects of GFRIPZ ranked in descending order are Zhejiang, Guangdong, Jiangxi, Guizhou, and Xinjiang. In addition, green finance has a more significant impact on heavy-polluting industries, large and state-owned enterprises, and listed companies located in the eastern region. Furthermore, the effects of industry heterogeneity ranked in descending order are energy, manufacturing, processing, and engineering industry, while it is not obvious in the service industry. Mechanism analysis suggests that the effect is driven by a reduction in the cost of debt financing and an increase in the long-term debt ratio. The findings provide implications for policymakers to promote the level of green innovation and environmental governance. Therefore, policymakers should support the long-term creative development of green invention patents by reducing the cost of debt financing and increasing the long-term debt ratio and consider the heterogeneous characteristics of listed companies when formulating green finance policies.

## 1. Introduction

With the carbon dioxide emissions in the world intensifying, environmental governance and green development have been the global consensus [1], which call for potent support from green finance policies [2]. In recent years, financial sectors have begun to pay attention to the relationship between green finance policies and sustainable economic growth [3]. It is believed that green finance policies can reduce carbon dioxide emissions by promoting green innovation of listed companies, which has been the best financial strategy for environmental governance [4]. The systematic argument for green finance originated from [5], which proposed that green finance incorporates environmental governance and conservation factors into investment and financing activities to achieve green allocation of funds. By tightening credit constraints on polluting listed companies while increasing financial support for environmentally friendly listed companies, green finance can facilitate the flow of capital from heavy-polluting industries to non-heavy polluting industries [6].

China has been the largest carbon emission country in the world, facing increasingly severe pressure from environmental pollution [7]. It is of urgent need for Chinese listed companies to enhance the green innovation level to reduce carbon emission and mitigate environmental pollution. In this context, the five provinces Zhejiang, Jiangxi, Guangdong, Guizhou, and Xinjiang were selected as the green finance reform and innovation pilot zone (GFRIPZ) by the State Council of China in June 2017. The contents and tasks for the establishment of the green finance reform and innovation pilot zone originate from the policy document promulgated by the State Council of China in 2017 [8]. This policy document states that the GFRIPZ was established to expand financial credit and provide low-interest and long-term loans to green listed companies, thereby allocating financial resources to environmentally friendly projects. Table 1 presents the specific contents and tasks of the construction of the green finance reform and innovation pilot zone.

**Figure 1:**
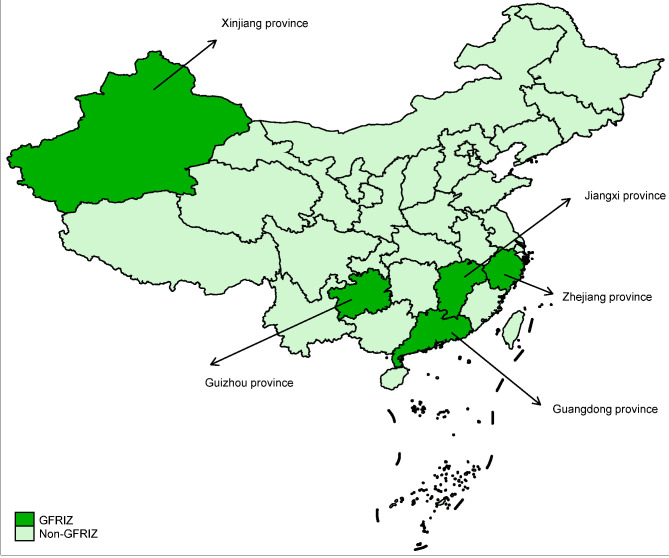
The implemented provinces of the GFRIPZ.

**Figure 2:**
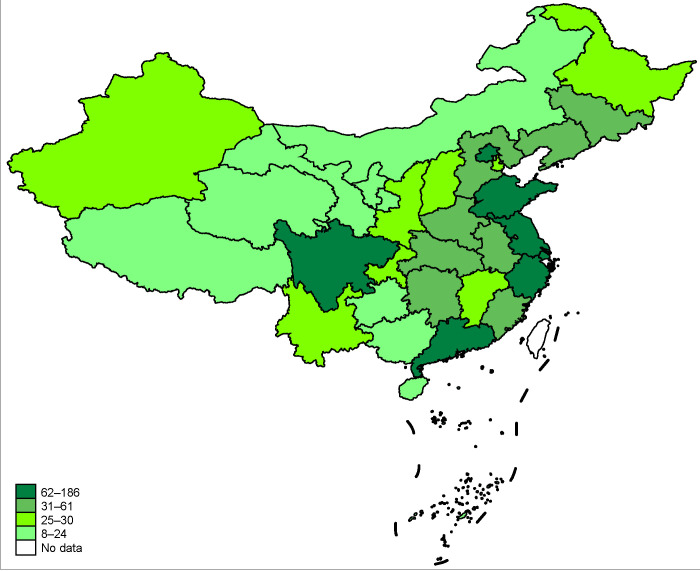
The amount of green loans in different provinces at the end of the year 2020.

By the end of 2020, the amount of green loans in the five provinces has reached 236.83 billion CNY, accounting for 15.1% of all loan balances, which is 4.3 percentage higher than China’s national average level. The balance of green bonds reached 135 billion CNY at the end of 2020, an increase of 66% year-on-year [9]. Figure 1 shows the specific implemented provinces of the GFRIPZ in China. The areas presented in dark green represent GFRIPZ provinces, and the light green represents non-GFRIPZ provinces. Figure 2 displays the amount of green loans in each of China’s provinces at the end of the year 2020. The color from light to dark refers to the amount of green loans from less to more.

However, current financial support for green technology innovation is still insufficient relative to the goal of carbon neutrality. Green technology innovation projects are characterized by long investment cycles, uncertain returns, imperfect information disclosure mechanisms, and high evaluation costs, all of which discourage financial institutions from providing more loans to green projects [10]. Therefore, how to improve the green innovation level of listed companies has been a decisive factor in getting sufficient credit support. Not only should companies strengthen their own green innovation, but the government should also develop green financial policies and encourage green credit to support the improvement of green innovation [9]. As long as financial resources flow more to environmental protection and green industries, resources such as land and labor will be optimally allocated [11]. However, current research mostly fails to put forward the policy recommendations for governments to guide the financial resource flow to green firms and green projects, and to enhance the green innovation activities of listed companies. In view of the above research gap, an in-depth study that provides implications for policymakers and enterprises about how to improve green innovation level needs to be done.

Taking GFRIPZ as a quasi-natural experiment, this study analyzes the impact of green finance on the green innovation level of listed companies using the differences-in-differences (DID) model. In order to reflect the differences in the degree and value of green innovation, we further divide green patent applications into green invention patent applications and green utility model patent applications. Through relevant robustness tests, heterogeneity analysis, and mechanism analysis, we further examine the policy impact of the GFRIPZ and its mechanisms. The results of the study provide insights for the government to formulate green financial policies and promote green innovation and green transformation of enterprises, so as to achieve low-carbon economic development and mitigate environmental pollution.

The remainder parts of this manuscript are structured as follows. The Literature Review section summarizes the relevant studies. The data, variables, and empirical methods utilized in this study are described in the Materials and Methods section. Our empirical findings are presented in the Results section. The Discussion section discusses the results in an international context. In the Conclusions section, we draw conclusions from the empirical analysis, make relevant policy recommendations on how to improve green innovation of listed companies, and present the limitation and possible directions in the future.

## 2. Literature Review

This section divides the literature related to this study into the following two aspects: the first is the impact of green finance policy, and the second is the factors influencing the green innovation level of listed companies.

Green finance policy has been a useful complement to traditional environmental regulation policies, as green finance combines the dual characteristics of financial resource allocation and environmental regulation [9]. Green finance establishes green investment and financing incentives, internalizes environmental pollution into financing costs for enterprises that discharge pollution, promoting the flow of capital from heavily polluting industries to low-pollution industries [6]. The development of green finance has contributed to an increase in the return on investment and availability of capital for the green sector by increasing financial support for environmentally friendly listed companies and reducing the investment and availability of capital for heavily polluting industries [12].

In terms of the impact of green finance policies, Saeed Meo and Karim [4] examined the relationship between green finance and carbon dioxide (CO_2_) emissions in the top ten economies that support green finance, including Canada, Denmark, Hong Kong, Japan, New Zealand, Norway, Sweden, Switzerland, the United Kingdom, and the United States. It is confirmed that green finance is the best financial strategy to reduce CO_2_ emissions. Al Mamun et al. [13] studied the effect of green finance on decarbonization using a large sample of 46 countries. The research shows that green finance significantly reduces carbon emissions both in the short and long run. This effect is driven by green bonds to control waste and pollution emission and improve energy efficiency. Mzoughi et al. [14] aim to build an incentive mechanism to mobilize the financial resources to accelerate the transition toward a climate-resilient society. Using VARs and COVARs, they found that green instruments (mainly the green bonds) are significantly affected by substantial price spillovers from the energy commodity market during critical periods. Some scholars have found that green finance has a positive effect on carbon emission reduction and have explored various tools of green finance [15]. Beck [16] argued that green financial products such as green funds and green insurance can drive private capital into the energy conservation and environmental protection sector. Li and Hu [17] concluded that the participation in green insurance helps listed companies to strengthen environmental risk management. Besides, many studies focus on the policy effects of green credit. Lian [18] found that green credit effectively promoted the development of green listed companies, by comparing the debt financing costs of green listed companies with those of high energy-consuming and heavily polluting listed companies. Wang et al. [12] focused on the impact mechanism of green financial development on the investment of green listed companies and found that green financial development could not only produce direct investment growth effects to promote green corporate investment, but also alleviate the maturity mismatch problem through the bias effect on the maturity structure of corporate debt and, thus, generating indirect investment growth effects. Liu et al. [19] found that green credit policies could have a significant financing penalty effect and investment disincentive effect on high energy-consuming and heavily polluting listed companies. Su and Lian [20] comprehensively examined the impact of green credit on the investment and financing behavior of heavily -polluting listed companies and found that the interest-bearing debt and long-term liabilities decreased significantly and the largest decrease was observed in large state-owned listed companies in high-emission areas. Some studies further found that green credit promoted corporate R&D investment and eco-innovation, technological innovation, and industrial structure upgradation [21,22,23]. Furthermore, some literature argued that direct financing represented by green securities is more effective for the development of environmental listed companies than indirect financing represented by green credit [24]. However, no uniform conclusion has been reached on whether green finance is effective in reducing corporate financing costs and improving economic returns [25,26].

From the perspective of the green innovation level, Rennings [27] provides a representative definition of green innovation that considers green innovation as a new idea, product, service, process, or management system to address environmental issues. Green innovation is both “green” in terms of social responsibility and an “innovation” in economic development, which not only solves environmental problems and improves environmental performance, but also enhances the competitiveness of companies by reducing production costs and differentiating their operations [28]. The existing literature mainly focuses on the impact of environmental regulation on green innovation [29,30], or the impact of green credit on the green innovation of listed companies [31,32]. Some scholars have also studied the relationship between green innovation level and corporate governance [33,34,35,36,37]. Three main views have emerged on the impact of environmental policies on green technology innovation: First, environmental regulation promotes green technology innovation. Different forms of environmental regulation have different effects on green technology innovation [38]. Market-inspired environmental regulation is more likely to promote green innovation by firms [30]. Second, environmental regulation inhibits green technology innovation. The strength of environmental regulation is negatively correlated with the number of patents related to green technology innovation [39]. Third, there is a non-linear relationship between environmental regulations and green technology innovation [40]. Some scholars found that government environmental regulations promoted green technology innovation [41,42,43]. The institutional pressure promoted listed companies to increase green innovation-related R&D investment and promote green innovation activities. Qi et al. [34] investigated whether the environmental rights trading market induced green innovation among firms. It was found that emissions trading policies in pilot regions induced green innovation activities among firms in heavily polluting industries compared to non-pilot regions and clean industries. Brunnermeier et al. [44] found that the higher the firm’s investment in environmental management, the more amount of green patent applications. Zhang et al. [45] found that R&D investment has a significantly positive effect on green technology innovation and the effect increases with time passed, while the effect of environmental regulation is stronger in the short term compared to the effect of R&D investment. This effect changes over time to be significantly lower than the positive effect of R&D investment. Additionally, executive characteristics such as environmental awareness, education level, gender, and employment background may also have positive or negative impact on corporate green innovation [46,47,48].

To sum up, the existing studies have the following limitations. First, most studies fail to combine the green finance and green innovation levels of listed companies. There is a lack of empirical studies on the impact of green financial policies on the level of green innovation of listed companies and their internal mechanisms. Second, most of the existing empirical studies on the impact of green finance on listed companies have been conducted from a single green financial instrument and product. Contrary to that, the establishment of the GFRIPZ is a comprehensive green finance policy that marks the encouragement of pilot provinces to establish a thorough green financial system. Third, although the establishment of the GFRIPZ has attracted attention from some scholars [10,49,50,51], the previous studies fail to use comprehensive indicators to measure the effect of it. They generally use the single metrics to measure green innovation levels, which are deficient in the scope of empirical analysis.

## 3. Materials and Methods

### 3.1. Variables and Data

This study selected the data of A-share listed companies in China from 2008 to 2020. We obtained the international patent classification (IPC) number of A-share listed companies from the Chinese Research Data Services Platform (CNRDS) [52]. Then we matched IPC number with the IPC Green Inventory issued by the World Intellectual Property Organization (WIPO) in 2010 [53]. If IPC number of a patent application was within IPC Green Inventory, it was identified as a green patent application, otherwise it was a non-green patent application. This method is similar to the studies of [34,35,36,54]. Furthermore, we used three metrics including green patent applications, green invention patent applications, and green utility model patent applications to measure the green innovation level [33,55]. To obtain robust regression results, we added one to the above three measurements and took the natural logarithm, consequently obtained Grepatent, Greinva, and Greuma, respectively, which were taken as the explained variables in the empirical analysis [29,55]. Control variables were selected in this study by drawing on studies of [9,31,56]. The data of control variables and mechanism variables come from the CSMAR database [57]. Variable definitions and descriptive statistics are shown in Table 2. To exclude the disturbance of extreme values, all variables were winsorized by 1%. The following samples were excluded from this study: ST samples, ST* samples, and PT samples. The total number is 1558, while the companies in the pilot zones are 386. Specifically, there are 123 companies in the Zhejiang Province, 27 companies in the Jiangxi Province, 186 companies in the Guangdong Province, 19 companies in the Guizhou Province, and 31 companies in the Xinjiang Province. Since the sample period was from 2008 to 2020, the observations are 20,254 in total, while the observations in the pilot zones are 5018.

### 3.2. Methods and Model Design

In order to analyze the impact of green finance policies on the green innovation of listed companies, we adopted China’s GFRIPZ of 2017 to construct the DID model following the methods and models of [9,29].

(1) $$\;Innovation_{i,p,t}=\beta_{0}+\beta_{1}D_{it}+\sum \gamma_{k}x_{kit}+\mu_{i}+\delta_{p}+\lambda_{t}+\varepsilon_{i,p,t}$$

In model (1), *i*, *p*, and *t* represent listed companies, provinces, and years, respectively. $Innovation_{i,p,t}$ represents the green innovation level of listed companies, measured by three metrics including $\;GreTotal_{i,p,t}\;,\;GreInva_{i,p,t}\;,\;\mathrm{and}\;GreUma_{i,p,t}$ in the empirical analysis. $D_{it}$ is the interaction term between $treat_{i}$ and $post_{t}$., which is policy dummy variable in the DID model.$\;Treat_{i}$ is the dummy variable of the pilot areas of GFRIPZ. Hence, listed companies in the five provinces of Zhejiang, Jiangxi, Guangdong, Guizhou, and Xinjiang are selected as the treatment group, otherwise the control group. $Treat_{i}$ of treatment group is taken as 1, otherwise $treat_{i}\;$= 0. $Post_{t}$ is the time dummy variable. In 2017 and later, $post_{t}$ = 1, otherwise $post_{t}\;$= 0. $\beta_{1\;}$is the core indicator to measure the policy effect. If $\beta_{1}$ is significantly positive, it is indicated that GFIFPZ has promoted the green innovation level of listed companies. $x_{kit}\;$represents control variables. We control firm-fixed effect $\mu_{i}$, province-fixed effect $\delta_{p}$ and year-fixed effect $\lambda_{t}$ in the empirical regression. $\varepsilon_{i,p,t}$ represents the random error term.

## 4. Results

### 4.1. Benchmark Regression Analysis

This study selected Grepatent, Greinva, and Greuma as explained variables to conduct regression using model (1), respectively. The results of benchmark regression analysis are presented in Table 3. We used Grepatent as the explained variable in columns (1)–(2), Greinva as the explained variable in columns (3)–(4), Greuma as the explained variable in columns (5)–(6) for regression analysis. The difference between columns (1), (3), (5) and columns (2), (4), (6) is whether control variables are added or not. It can be seen that whichever measurements of green innovation level that are used in the regression, the coefficients of $\;D_{it\;}$ are all positive. However, the significance of $\beta_{1}$ is different among these measurements. The coefficients of columns (1)–(2) are both significant at the 5% level. The coefficient of $\;D_{it\;}$column (3) is not significant. However, in column (4), the coefficient of$\;D_{it\;}$is significant at the 10% level. In addition, the coefficients of $\;D_{it\;}$in columns (5)–(6) are significant at the 5% and 1% levels, respectively. The results indicate that the GFRIPZ has promoted the green innovation level of listed companies, especially for the improvement of green utility model patent applications. Comparatively, the positive effect on green invention patent applications is less obvious. This may be due to the lower level of creativity required for a green utility model patent application than a green invention patent application and an easier approval process (the differences between green utility model patent applications and green invention patent applications are presented in Supplementary Document, as shown in Table S1). With the support of green finance, listed companies give priority to applying for green utility model patents, thus delaying the invention and application of green invention patents.

In order to present the influence of the volume of green finance, we used a continuous DID model by introducing the interaction term between Green and post in this section. We replaced the dummy variable of whether the place implemented GFRIPZ or not (Treat) with the volume of green loans (Green). It means that $D_{it}\;$in model (1) is replaced by the interaction term between Green and post (Treat × post → Green × post). It can be seen from Table 3 that, in terms of the influence of total volume of green loans, green finance volume has a positive impact on the green innovation of listed companies. Specifically, the increase in the applications of green utility model patent is pronounced, while the effect on green invention patent applications is not obvious. The green innovation level of listed companies is indeed affected by green finance, not just because they are registered in the region that happens to be a pilot zone.

**Figure 3:**
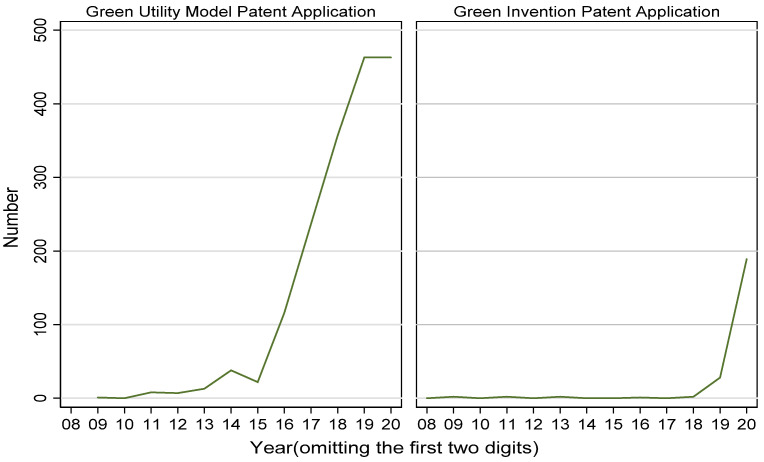
The trend of the number of green invention patent applications and green utility model patent applications over the years.

Figure 3 shows the trend of the number of green invention patent applications and green utility model patent applications over the years. The vertical axis stands for the number of green invention patent applications and green utility model patent applications. The horizontal axis represents the year, using abbreviations: 20 for 2020, 19 for 2019, and so forth. As shown in Figure 3, after the implementation of the GFRIPZ, the number of green utility model patent applications surged, and the growth rate of green invention patent applications has been far less than that of green utility model patent applications. It verifies the conclusion of benchmark regression.

### 4.2. Robustness Test

This study uses four methods to analyze the robustness of the regression results and verify the accuracy of the empirical conclusions: the first method is the parallel trend test, the second method is the placebo test, the third method is PSM-DID estimation, and the fourth method is the replacement of the explained variables [11,49,58].

#### 4.2.1. Parallel Trend Test

Conforming to the parallel trend assumption is an important prerequisite of the DID model. Parallel trend means that the treatment group and the control group have the same time trend before the implementation of the GFRIPZ. The parallel trend before implementation of the GFRIPZ and the dynamic effect after adopting the policy are estimated by the following model by drawing on the model construction of [59].

(2) $$\;Innovation_{i,p,t}=\beta_{0}+\beta_{i}\sum_{k=-3}^{3}D^{k}{}_{it}+\sum \gamma_{k}x_{kit}+\mu_{i}+\delta_{p}+\lambda_{t}+\varepsilon_{i,p,t}$$

**Figure 4:**
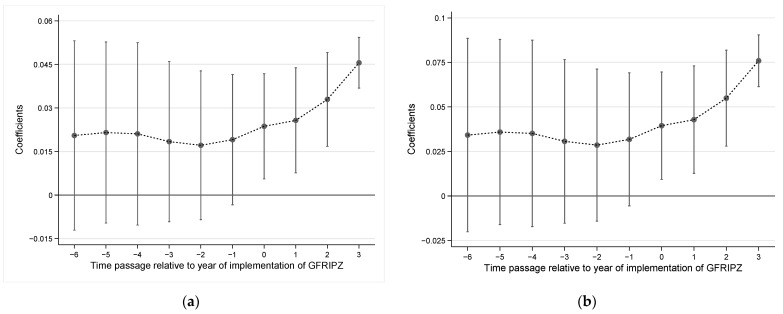
Parallel trend test: (**a**) Without control variables; (**b**) With control variables.

In model (2), *k* represents time passage relative to the year of implementation of the GFRIPZ, which ranges from −3 to 3. The value of *k* is 0 in 2017. In *k* years after the adoption of the GFRIPZ among the treatment group, the value of $D^{k}{}_{it}$ is 1, otherwise the value of $D^{k}{}_{it}$ is 0. Based on the estimated coefficient $\beta_{i}$ obtained from the regression, Figure 4a,b are plotted. This figure includes (a) without control variables and (b) with control variables. The horizontal axis indicates time passage relative to year of implementation of the policy *k*, and the vertical axis represents the estimated coefficients $\beta_{i}$. It can be shown that no matter whether the control variables are added, there is no obvious trend difference in green innovation level between treatment group and control group before the implementation of the GFRIPZ. Instead, after the implementation of the GFRIPZ, green patent applications of listed companies began to increase significantly. It indicates that green finance policy has a significant effect on promoting the level of green innovation of listed companies. To sum up, the parallel trend assumption is satisfied.

#### 4.2.2. Placebo Test

**Figure 5:**
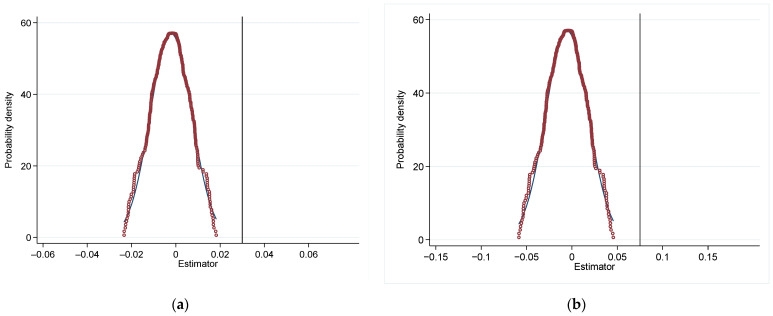
Placebo test: (**a**) Without control variables; (**b**) With control variables.

To avoid that the baseline results are accidental or come from other unobservable factors, we conducted a placebo test to verify the results [11]. The core idea of the placebo test is to estimate the benchmark regression model based on the fictitious treatment group [60]. We randomly selected some companies from the samples as the fictitious treatment group and re-estimated model (1). This process was repeated 1000 times and the estimated coefficients of $D_{it}$ in the fictitious treatment group could be obtained. The kernel density plot is drawn as shown in Figure 5. It includes (a) without control variables and (b) with control variables. The horizontal axis represents the estimated coefficient of $D_{it}$ obtained from the fictitious treatment group, and the vertical axis indicates probability density. The vertical line is the true value of the estimated coefficient of $D_{it}$ in the benchmark regression. And the red curve represents the probability density of each estimated value. It can be found that the estimated coefficients of $D_{it}$ are significantly centered around 0 and different from the true values (0.031 without control variables, 0.073 with control variables), regardless of whether control variables are added or not. It is suggested that the effects of the GFRIPZ on the green innovation of listed companies are not accidental or stem from other unobservable factors, but are actually caused by the implementation of the green finance policy itself.

#### 4.2.3. PSM-DID Estimation

This section further uses the difference-in-difference estimation after propensity score matching (PSM-DID) for robustness test. There are usually three matching methods, namely kernel matching, caliper matching, and nearest neighbor matching [49,58,61]. The specific steps are as follows. Firstly, the logit regression model was constructed using the control variables in the benchmark model as the matching feature variables. Secondly, the predicted values of the logit model were used as the matching scores, and we excluded the samples that were not successfully matched. Thirdly, we used the DID model for regression, based on the data obtained after kernel matching, caliper matching, and nearest neighbor matching. Table 4 presents the results of the PSM-DID estimation. We used kernel matching in columns (1)–(3), caliper matching in columns (4)–(6), and nearest neighbor matching in columns (7)–(9). All regressions include control variables. The results show that of $\;D_{it\;}$ are positive across the board regardless of kernel matching, nearest neighbor matching, or caliper matching adopted for PSM-DID estimation. Besides, the coefficients $\beta_{1}$ of columns (2), (5), (8) are not significant, whereas the coefficients in other columns pass the significance test. Overall, the results are consistent with those of the benchmark regression, so the regression results are robust.

#### 4.2.4. Replace the Explained Variables

Considering that relative indicators of green patents may be more effective than absolute indicators in eliminating unobservable factors outside a certain policy [34]. This section replaces the number of green patent applications (Grepatent) with the proportion of green patent applications to all patent applications (Rpatent), the number of green invention patent applications (Greinva) with the proportion of green invention patent applications to all invention patent applications (Rinva), the number of green utility model patent applications (Greuma) with the proportion of green utility model patent applications to all utility model patent applications (Ruma) [9]. The descriptive statistics of the surrogate variables are shown in Table 5.

Table 6 shows the results of replacing the explained variables. It can be seen that the coefficients of$\;D_{it\;}$are 0.021 and 0.014 in columns (1) and (2), respectively. $\beta_{1\;}$is significant at the 5% level when no control variables are added, while significant at the 10% level when control variables are added. In columns (3) and (4), the coefficients of$\;D_{it\;}\;$are 0.031 and 0.032, respectively, both of which are not significant. In columns (5) and (6), $\beta_{1\;}$are 0.027 and 0.043, respectively, and both are significant at the 5% level. The results show that the implementation of the GFRIPZ has a positive impact on the green innovation level of listed companies, especially increasing green utility model patent applications, while the positive effect on green invention patent applications is not pronounced. It is consistent with the results in Table 3, indicating that the regression conclusions are accurate.

### 4.3. Heterogeneity Analysis

#### 4.3.1. Heterogeneity Analysis of Different Pilot Zones

In view of the different goals and tasks of the five pilot zones, there are differences in the effect of green finance on the green innovation level among different pilot zones. This study uses Grepatent as the explained variable for heterogeneity analysis. The results of heterogeneity analysis of different pilot zones are shown in Table 7. It can be seen that the coefficient of $D_{it\;}$ in the pilot zone of Zhejiang is 0.263, which performs best among the five pilot zones. Guangdong, Jiangxi, and Guizhou are followed by the Zhejiang pilot zone in order of performance, of which the coefficients are 0.158, 0.139, and 0.116, respectively. The Xinjiang pilot zone performs worst, and the coefficient is 0.081.

During the implementation of the GFRIPZ, the volume of green finance in different pilot zones has been different and increased year by year. In order to overcome the limitation that our research merely focuses on the companies registered in the region that happens to be a pilot zone, we further demonstrate the influence of the volume of green finance in each pilot zone. We used continuous DID model by introducing the interaction term between Green and post in this section, which was also used in Section 4.1. It can be seen from Table 7 that, in terms of the influence of green loans volumes in each pilot zone, green finance actually has a positive impact on green innovation of listed companies. It indicates that the green innovation level of listed companies is indeed affected by the green finance, not just because that they are registered in the region that happens to be a pilot zone. Furthermore, we can conclude that among all these pilot zones, the policy effects of the GFRIPZ ranked in descending order are Zhejiang, Guangdong, Jiangxi, Guizhou, and Xinjiang.

#### 4.3.2. Heterogeneity Analysis of Listed Company Characteristics

Some studies concluded that differences in the effect of green finance policies on green innovation level of firms are caused by the following listed company characteristics, including ownership attributes of the listed companies, pollution degree of the industry that the listed companies belong to, the scale of the listed companies, and the region where the listed companies are located [9,31,50]. By drawing on the above studies, this section introduces the interaction term$\;D_{it\;}\times Owner_{it\;}\;,\;D_{it\;}\times Industry_{it\;},\;D_{it\;}\times Scale_{it\;},\;\;D_{it\;}\times Region_{it\;}$in model (1), respectively, in order to test whether the impact of green finance policies on green innovation of listed companies has the above heterogeneous characteristics. The triple-differences models are constructed as follows [11].

(3) $$\;Innovation_{i,p,t}=\beta_{0}+\beta_{1}D_{it}+\beta_{2}(D_{it\;}\times Owner_{it})+\sum \gamma_{k}x_{kit}+\mu_{i}+\delta_{p}+\lambda_{t}+\varepsilon_{i,p,t}$$

(4) $$Innovation_{i,p,t}=\beta_{0}+\beta_{1}D_{it}+\beta_{2}(D_{it\;}\times Industry_{it})+\sum \gamma_{k}x_{kit}+\mu_{i}+\delta_{p}+\lambda_{t}+\varepsilon_{i,p,t}$$

(5) $$\;Innovation_{i,p,t}=\beta_{0}+\beta_{1}D_{it}+\beta_{2}(D_{it\;}\times Scale_{it})+\sum \gamma_{k}x_{kit}+\mu_{i}+\delta_{p}+\lambda_{t}+\varepsilon_{i,p,t}$$

(6) $$\;Innovation_{i,p,t}=\beta_{0}+\beta_{1}D_{it}+\beta_{2}(D_{it\;}\times Region_{it\;})+\sum \gamma_{k}x_{kit}+\mu_{i}+\delta_{p}+\lambda_{t}+\varepsilon_{i,p,t}$$

In model (3), model (4), model (5), and model (6), $Owner_{it\;},Industry{\;}_{it\;},\;Scale_{it\;}\;and\;Region_{it\;}$are the grouping dummy variables. There are four classifications for the entire sample. The samples are divided into state-owned listed companies and non-state-owned listed companies by introducing $\;Owner_{it\;}$. State-owned enterprises have the value of 1 and non-state-owned enterprises have the value of 0. Moreover, we divided the samples according to the pollution degrees of industries that the listed companies belong to. Referring to the study of Pan et al. [62], whether a listed company belongs to heavily polluting industries or not was sifted out by comparing the Guidelines for Industry Classification of Listed Companies [63] and the List of Industry Classification Management of Listed Companies for Environmental Protection Verification [64]. The number of companies belonging to heavily polluting industries is 607, while non-heavy polluting companies is 951. When an enterprise belongs to the heavily polluting industry, the value of $\;Industry_{it}$ is 1, otherwise the value of $\;Industry_{it}$ is 0. Besides, we divide the samples into large-scale listed companies and small-scale listed companies according to the 50th percentile of total assets of companies [9]. Large-scale enterprises have the value of 1. Small-scale enterprises have the value of 0. We also classified the samples into eastern region, central and western regions in accordance with the region where listed companies are located. The central and western provinces in China are further merged according to the study of [65]. If listed companies are located in the eastern region, the value of $\;Region_{it\;}$ is 1. However, if the listed companies are located in the central and western regions, the value of $\;Region_{it\;}$ is 0.

The results of heterogeneity analysis of listed company characteristics are shown in Table 8. The coefficient of $D_{it\;}\times Owner_{it\;}$ is significantly positive, indicating that the GFRIPZ promotes green innovation of state-owned listed companies more obviously, compared with non-state-owned listed companies. The coefficient of $D_{it\;}\times Industry{\;}_{it\;}$ is significantly negative, which suggests that green innovation of heavily polluting industries is more affected by green finance policies than non-heavy polluting industries. The reason is that green finance is used to restrict the capital allocation of heavily polluting industries through financial instruments, guiding resources away from highly polluting outdated production capacity. It not only restrains the financing of heavily polluting enterprises, but more importantly, forces the transformation and upgrading of heavily polluting enterprises to get rid of a traditional production mode that consumes much more energy and causes higher pollution. However, non-heavily polluting industries are not the focus of green finance policy. When a heavily polluting company faces the financing constraints brought by the green financial policy, on the one hand, it is the reaction of passively reducing capital investment, and on the other hand, it is manifested as the strategic behavior of improving total factor productivity through technological innovation. Furthermore, green finance has more effective improvement on the green innovation level of large-scale listed companies than small-scale listed companies according to significantly positive coefficient of $D_{it\;}\times Scale_{it\;}.\;$In addition, the coefficient of $D_{it\;}\times Region_{it\;}$ is 0.309 at the 5% significance level, suggesting that the GFRIPZ has a more obvious effect on promoting green innovation activities of listed companies in the eastern region than those in the central and western regions.

#### 4.3.3. Heterogeneity Analysis of Specific Industry Qualifications

Apart from the industry qualification of heavily polluting and non-heavily polluting, we conduct the heterogeneity analysis of specific industry qualifications including services, manufacturing, energy, processing, and engineering, in this section. The results of heterogeneity analysis of different pilot zones are shown in Table 9. It can be found that the coefficient of $D_{it\;}$ in energy industry is the largest, which is significantly estimated to be 0.277 at the 1% significance level. The coefficient in manufacturing, processing, and engineering industry are 0.202, 0.186, and 0.094, respectively, which all passed the significance test. In the services industry, the coefficient of $D_{it\;}$ is the smallest, which is not significant. It can be concluded that the effects of the GFRIPZ on the green innovation of listed companies in the energy, manufacturing, processing, and engineering industries are in a descending order, while the effect is not obvious in the services industry.

### 4.4. Mechanism Analysis

Green finance provides lower financing costs and long-term funds for green innovation activities of listed companies [6]. Listed companies can not only obtain loans with lower interest rates to reduce financing costs and invest more funds in green projects, but also obtain more long-term capital borrowing to improve the debt structure of listed companies [9; 29]. Therefore, this study selects debt financing cost (Debt) and long-term debt ratio (LDR) as mediating variables to test the internal mechanism of the impact of the GFRIPZ on the green innovation level of listed companies. This section adopts a stepwise regression test for coefficients to analyze the mediation effect [66,67]. Model (7), model (8), and model (9) are constructed as mediation effect models for regression.

(7) $$\;Innovation_{i,p,t}=\alpha +cD_{it}+\sum \delta_{1k}x_{kit}+\mu_{i}+\delta_{p}+\lambda_{t}+\varepsilon_{i,p,t}$$

(8) $$M_{i,p,t}=\alpha +aD_{it}+\sum \delta_{2k}x_{kit}+\mu_{i}+\delta_{p}+\lambda_{t}+\varepsilon_{i,p,t}$$

(9) $$\;Innovation_{i,p,t}=\alpha +c^{’}D_{it}+bM_{i,p,t}+\sum \delta_{3k}x_{kit}+\mu_{i}+\delta_{p}+\lambda_{t}+\varepsilon_{i,p,t}$$

Among them, $M_{i,p,t}$ is the mediating variable, including debt financing cost (Debt) and long-term debt ratio (LDR). c represents the total effect of GFRIPZ. c’ represents the direct effect. ab represents the indirect effect, namely the mediating effect. α represents the constant. The definitions and descriptive statistics of mediating variables are presented in Table 10.

This study tested the coefficient c through model (7) in column (1). Columns (2)–(3) use debt financing cost (Debt) and long-term debt ratio (LDR) as mediating variables, respectively, to test the coefficient a in model (8). Columns (4)–(5) use debt financing cost (Debt) and long-term debt ratio (LDR) as mediating variables, respectively, and conduct regression based on model (9) to obtain coefficients c’ and b. We selected Grepatent as the explained variable for mechanism analysis. Table 11 presents the results of stepwise regression test for coefficients. It can be seen that the coefficients *a*, *b*, *c* are all significant, so the mediation effect could be effective [67]. In addition, we find that the coefficients of columns (2) and (4) are significantly negative, −0.034 and −0.225, respectively, while the coefficients of columns (3) and (5) are significantly positive, 0.093 and 0.104, respectively. On the one hand, the GFRIPZ has reduced debt financing cost. Financial institutions have lower loan costs for corporate green projects. Therefore, listed companies are willing to borrow more low-interest funds to invest in the development of green technology innovation. On the other hand, the GFRIPZ has increased the proportion of long-term debts of listed companies, providing more long-term funds for companies to improve the debt structure. Therefore, listed companies can invest more funds into the invention and applications of green patents, which promotes the green innovation level.

## 5. Discussion

This study makes an important contribution in exploring the policy effects of green financial reform and innovation pilot zones on the level of green innovation of listed companies and their intrinsic impact mechanisms. Using the GFRIPZ established in China in 2017 as a quasi-natural experiment, this paper successively adopted the DID model, PSM-DID estimation, the triple-differences model, and the mediating effect model to test whether green finance policy improves the green innovation level of listed companies and mechanism paths. This study not only enriches the research on green financial policies and the level of green innovation, but also provides insights for policy makers to formulate policies to promote green innovation in listed companies for the purpose of low-carbon economic development and environmental protection.

The research object in this paper is similar to that of Zhang and Li [51], but the empirical method, variable selection, and mechanism paths are quite different. They used the difference-in-difference method to test the impact of the green finance reform and innovation pilot zone on green innovation activities. However, this paper employs more different methods in the empirical analysis, such as PSM-DID estimation, the continuous DID model, and the triple-difference model. PSM-DID estimation is used as one of the robustness test methods, and the triple-difference model is used to analyze the heterogeneity characteristics of the policy effects of the GFRIPZ, including ownership attributes of listed companies, pollution degree of the industry that listed companies belong to, the scale of listed companies, and the region where listed companies are located. We also conducted a heterogeneity analysis of different pilot zones, and a heterogeneity analysis of specific industry qualifications. The continuous DID model is used for presenting the volume of green finance, both in benchmark regression and heterogeneity analysis of different pilot zones. In the study of [51], the heterogeneity analysis selected sample-divided regression without introducing a triple-difference interaction term. In the mechanism analysis, our study selected debt financing cost (Debt) and long-term debt ratio (LDR) as mediating variables to test the internal mechanism of the GFRIPZ using stepwise regression test for coefficients, which is quite different from the research of Zhang and Li [51]. In their study, R&D investment (R&D) and credit size (Debt) are selected as mediating variables. They argue that the pilot policy promotes the quantity and quality of green innovation by increasing the R&D investment and credit expansion of firms in the pilot region. However, we conclude that the GFRIPZ increases the green innovation level of listed companies by reducing the cost of debt financing and enhancing long-term debt ratio.

Green finance is not only thriving in China, but also happening around the world. From an international perspective, the conclusions of Saeed Meo and Karim [4] and Al Mamun et al. [13] are supported by the findings in this paper. Saeed Meo and Karim [4] argued that green finance is the best financial strategy to reduce CO2 emissions, which is conducive to environmental protection. It is confirmed by our finding that green finance can promote green innovation levels, thus reducing environmental pollution. Al Mamun et al. [13] concluded that green finance significantly reduces carbon emissions both in the short and long run and this effect is driven by green bonds to control waste and pollution emission and green innovation of enterprises to improve energy efficiency, which can be also confirmed in our study. Although the research perspective of our study is different from Mzoughi et al. [14] and Amore and Bennedsen [33], the issues we pay attention to are the same. Some implications for policymakers to promote the implementation of green finance strategy, green innovation of listed companies, and environment protection are put forward in our research and theabove-mentioned studies.

## 6. Conclusions

Based on the panel data of A-share listed companies in China from 2008 to 2020, this study used the DID model to analyze the impact of green finance policy on green innovation level of listed companies and its mechanism by taking the GFRIPZ implemented in 2017 as a quasi-natural experiment. The results show that the GFRIPZ improves the green innovation level of listed companies, especially increasing green utility model patent applications, while positive effect on green invention patent applications is less obvious.

Among all these pilot zones, the policy effects of the GFRIPZ on green innovation of listed companies in Zhejiang, Guangdong, Jiangxi, Guizhou, and Xinjiang are in a descending order. Furthermore, the GFRIPZ promotes green innovation of state-owned listed companies more obviously, and green innovation of heavily polluting industries is more affected by green finance policies than non-heavily polluting industries. Green finance has more pronounced improvement on the green innovation level of large-scale listed companies than small-scale listed companies. In addition, the GFRIPZ has a more significant effect on promoting green innovation activities of listed companies in the eastern region than those in the central and western regions. In terms of specific industry, the effects of the GFRIPZ on green innovation of listed companies in energy, manufacturing, processing, and engineering industry are in a descending order, while the effect is not obvious in the services industry. The mechanism analysis indicates that the effects are caused by decreased debt financing cost and elevated long-term debt ratio. On the one hand, the GFRIPZ has reduced debt financing cost. Financial institutions have lower loan costs for corporate green projects. Therefore, listed companies are willing to borrow more low-interest funds to invest in the development of green technology innovation. On the other hand, the GFRIPZ has increased the proportion of long-term debts of listed companies, providing more long-term funds for companies to improve the debt structure. Therefore, listed companies can invest more funds into the invention and applications of green patents, which promotes the green innovation level.

After the establishment of the green finance reform and innovation pilot zone, the support for green projects of listed companies has achieved obvious results. However, there is still a long way to go to further improve the construction of a green financial system and effectively support green technology innovation. Based on the above empirical analysis and findings, this paper puts forward the following recommendations about how to improve the green innovation of listed companies.

First, the study shows that Chinese listed companies do not pay attention to the creation of green invention patents, resulting in the application of green invention patents lagging far behind that of green utility model patents. This problem in China should be emphasized by the government. Policymakers should support long-term creative development of green invention patents by reducing debt financing cost and enhancing long-term debt ratio of listed companies, rather than merely focusing on short-term quick applications of green utility model patents.

Second, governments need to consider the heterogeneous characteristics of listed companies when formulating green finance policies. Authorities should implement differentiated green finance policy according to the pollution degree of the industry, the ownership attributes of the enterprise, the scale of the enterprise, and the region where the enterprise is located. More low-interest credit, long-term loans, and innovation incentives should be placed on state-owned companies, heavily polluting companies, large-scale companies, and listed companies in the eastern region.

Third, more attention should be paid to the preferential financing costs. The government should provide certain guidance and incentives for companies, including incubation, guarantees, and interest subsidies, in order to reduce the financing costs and risk premiums for green technology companies. Furthermore, authorities should provide support and tax incentive to encourage enterprises to increase the long-term debt ratio. Therefore, by reducing the cost of debt financing and increasing the long-term debt ratio, the investment in the application and invention of green patents can be increased, thus promoting the level of green innovation in listed companies.

It is inevitable that this paper has some limitations. First of all, we failed to examine other mechanism paths in the mediating effect of the GFRIPZ on the green innovation level of listed companies, merely selecting two mediating variables: debt financing cost (Debt) and long-term debt ratio (LDR). It is worth taking into account other mediating variables in future research. Furthermore, we failed to control the impact of other green policies promulgated by local governments on the green innovation of listed companies. Many other pilot zones might have different compounding factors that drive the green innovation of companies, which is a difficulty that we are not yet able to overcome. It provides an opportunity for the future to find a way to effectively control other factors except for the GFRIPZ that affect the green innovation of companies.

## Figures and Tables

**Table 1:** The construction contents and tasks of GFRIPZ.

GFRIPZ	Details in Contents and Tasks of GFRIPZ
Contents	In June 2017, Zhejiang, Jiangxi, Guangdong, Guizhou, and Xinjiang were selected as the green finance reform and innovation pilot zone.The authorities will step up efforts to motivate the financial institutions on the issuance of green credit, green insurance, and green bonds. A “one-vote veto” system for environmental protection has been implemented in the financing process. There are more long-term loans with low interest provided to green enterprises. Heavily polluting enterprises are restricted on the application of credit, and China has made further exploration on the establishment of environmental rights trading market. Enterprises in the pilot zones are encouraged to resolve excess capacity and eliminate backward production capacity through the limitation or withdrawal from high energy consumption, heavy pollution projects, and the establishment of a “white list” and a “black list” in the field of environmental protection.
**Each Pilot Zone**	**Details in Differences between the Pilot Zones**
Guangdong Pilot Zone	Guangdong will make more efforts to explore the establishment of a new development model in line with green financial reform and economic growth. According to the overall plan, the authorities will encourage the establishment of financial institutions that maintaining new energy vehicles, developing financial product innovations of new energy vehicles, issuing green bonds financing green, circular, and low-carbon projects. The Guangdong pilot zone will work on the following tasks, namely, cultivating the green financial organization system, supporting green industries to expand financing channels, building a trading market for green financial products, accelerating the development of green insurance, establishing a mechanism targeting green financial risk prevention.
Jiangxi Pilot Zone	By 2020, the growth rate of green credit balances in the pilot zone will be higher than the growth rate of other types of loans, and the proportion of green credit increments in total loan increments will be increased, according to the overall plan. The scale of direct financing, such as equity financing, is planned to expand continuously. By the end of 2020, the green credit volume of the pilot zone in Jiangxi will reach 300 billion CNY, accounting for a proportion of various loans that exceeds the national average, and the scale of green bond issuance will reach 30 billion CNY.
Zhejiang Pilot Zone	The scale of green finance is planned to attain a rapid growth in five years. The scale of financing for “high-pollution and high-emission” industries will decrease, and the non-performing loan ratio of green loans should not be higher than the average non-performing loan of small business loans. The Zhejiang pilot zone plans to build a green financial organization system, expanding financing channels for green industry, exploring and promoting the construction of an environmental rights trading market in a stable and orderly manner, developing green insurance, building a financial service mechanism for the transformation and upgrade of green industries, establishing a green financial system to support the development of small and medium-sized cities and featured small towns.
Guizhou Pilot Zone	According to the overall plan, Guizhou will establish a multi-level organizational system, a diversified product and service system, a multi-level support service system and an efficient and flexible market operation mechanism by 2022, and the scale of green credit issuance will increase. Practically, the pilot zone in Guizhou province will establish a multi-level green financial organization system to expedite the design of green financial products and services, to promote the benefits from energy-saving projects, government–private partnership projects (PPP), pollution discharge rights, and energy use rights. Efforts are needed to develop green credit products that benefit farmers and support agricultural industrial projects, such as organic ecological agriculture, rural water conservancy projects, and agricultural production sewage treatments. At the same time, local authorities should guide qualified green enterprises to issue green bonds, promote small and medium-sized green enterprises to issue green collective bonds, and explore the issuance of green project income bills.
Xinjiang Pilot Zone	It aims to increase the proportion of green credit, green bonds, green equity financing, etc., in the total financing, reduce the scale of loans to industries with “high pollution and high emissions” in about five years, and build a preliminary green financial system with regional characteristics, as stated in the overall plan. The pilot zone in the Xinjiang autonomous region will support financial institutions to carry out green finance strategies in line with financial institution headquarters or branches, to develop green credit products such as water-saving, energy-saving, and emission-reduction loans for green mine construction. More efforts are needed to support financial institutions, enterprises with long-term green projects to issue green bonds or project-supporting bonds, and to support heating supplemented by clean and renewable energy.

**Table 2:** Variable definitions and descriptive statistics.

Variable Types	Variable Statistics	Variable Definition	Observations	Means	Standard Deviation	Min	Max
Explained variables	Grepatent	Ln (number of green patent applications +1)	20,254	1.1707	1.6026	0	6.1399
	Greinva	Ln (number of green invention patent applications +1)	20,254	0.3371	0.7851	0	3.8918
	Greuma	Ln (number of green utility model patent applications +1)	20,254	1.1163	1.5611	0	6.0235
Explanatory variable	D	Policy dummy variable	20,254	0.0766	0.2660	0	1
Control variables	Top	Shareholding ratio of the largest shareholder	20,216	34.424	15.268	8.41	74.98
	Cash	Ending balance of cash and cash equivalents/current liability	20,237	0.5542	0.8313	0	5.524
	LAR	Ending balance of total liability/total asset	20,238	0.5629	0.2622	0.7775	1.6865
	RD	R&D cost/total expenditure	20,254	2.9253	3.4703	0	20.8
	ROE	The ratio of net income to total average equity	20,238	0.0414	0.2063	−1.3407	0.4132
	Size	Ending balance of total asset of listed companies	20,238	31	133	0.153	1170
	Tobin	(Market value of tradable shares + par value of non-tradable shares)/(total asset—net intangible asset—net goodwill)	18,132	2.1308	1.7796	0.8614	12.627
	Capital	The ratio of total asset to sales revenue	20,254	2.8947	3.9988	0.3483	29.803
	Ret	Annual return of individual shares	17,099	0.3976	1.7730	−0.7865	14.971

**Table 3:** Benchmark regression.

Variables	Grepatent		Greinva		Greuma	
	(1)	(2)	(3)	(4)	(5)	(6)
D	0.031 **	0.073 **	0.016	0.045 *	0.021 **	0.066 ***
	(0.051)	(0.090)	(0.031)	(0.055)	(0.049)	(0.088)
Green × post	0.069 * (0.237)	0.092 *** (0.028)	0.024 (0.016)	0.064 (0.042)	0.037 ** (0.034)	0.085 ** (0.062)
Top		−0.119 **		−0.164 *		−0.088
		(0.035)		(0.019)		(0.034)
Cash		0.043		0.032 *		0.042
		(0.032)		(0.017)		(0.031)
LAR		−0.051		−0.041		−0.028
		(0.159)		(0.087)		(0.153)
RD		0.021 **		0.015 ***		0.018 *
		(0.010)		(0.005)		(0.010)
ROE		0.144		0.066		0.137
		(0.097)		(0.049)		(0.095)
Size		0.507		0.326 *		0.496
		(0.109)		(0.022)		(0.058)
Tobin		0.056 ***		0.019 **		0.052 ***
		(0.015)		(0.008)		(0.014)
Capital		0.032 ***		0.016 ***		0.027 ***
		(0.011)		(0.005)		(0.010)
Ret		0.361 *		0.091 **		0.332 ***
		(0.079)		(0.042)		(0.079)
Constant	0.879 ***	1.490 ***	0.181 ***	0.347 ***	0.843 ***	1.394 ***
	(0.022)	(0.183)	(0.012)	(0.100)	(0.021)	(0.176)
Firm-fixed effect	Control	Control	Control	Control	Control	Control
Year-fixed effect	Control	Control	Control	Control	Control	Control
Province-fixed effect	Control	Control	Control	Control	Control	Control
Observations	20,254	17,099	20,254	17,099	20,254	17,099
R-squared	0.637	0.753	0.739	0.775	0.636	0.749

Notes: The parentheses are the clustered standard errors. ***, **, and * indicate significant at the 1%, 5%, and 10% levels, respectively.

**Table 4:** The results of PSM-DID estimation.

Variables	Kernel Matching			Caliper Matching			Neighbor Matching		
	(1)	(2)	(3)	(4)	(5)	(6)	(7)	(8)	(9)
D	0.027 **	0.313	0.016 **	0.041 ***	0.121	0.066 *	0.016 **	0.157	0.035 ***
	(0.145)	(0.120)	(0.031)	(0.008)	(0.047)	(0.088)	(0.011)	(0.026)	(0.077)
Control variables	Yes	Yes	Yes	Yes	Yes	Yes	Yes	Yes	Yes
Constant	1.748 ***	0.463 ***	1.672 ***	1.777 ***	0.445 ***	0.443 ***	1.701 ***	0.910 ***	1.779 ***
	(0.024)	(0.013)	(0.024)	(0.037)	(0.020)	(0.019)	(0.037)	(0.126)	(0.038)
Firm-fixed effect	Control	Control	Control	Control	Control	Control	Control	Control	Control
Year-fixed effect	Control	Control	Control	Control	Control	Control	Control	Control	Control
Province-fixed effect	Control	Control	Control	Control	Control	Control	Control	Control	Control
Observations	17,099	17,099	17,099	17,099	17,099	17,099	17,099	17,099	17,099
R-squared	0.705	0.689	0.786	0.653	0.658	0.638	0.757	0.824	0.726

Notes: The parentheses are the clustered standard errors. ***, **, and * indicate significant at the 1%, 5%, and 10% levels, respectively.

**Table 5:** Descriptive statistics of surrogate variables.

Statistic	Unit	Observations	Means	Standard Deviation	Min	Max
Rpatent	-	20,254	0.10812	0.20397	0	0.645
Rinva	-	20,254	0.88979	0.20598	0	0.321
Ruma	-	20,254	0.05511	0.08587	0	0.405

**Table 6:** Replacing the explained variables.

Variables	Rpatent		Rinva		Ruma	
	(1)	(2)	(3)	(4)	(5)	(6)
D	0.021 **	0.014 ***	0.031	0.032	0.027 **	0.043 **
	(0.010)	(0.019)	(0.028)	(0.017)	(0.028)	(0.036)
Top		−0.053		−0.429 ***		−0.442 ***
		(0.036)		(0.040)		(0.065)
Cash		0.032 ***		0.019*		0.022
		(0.017)		(0.011)		(0.035)
LAR		−0.070 ***		−0.042		−0.013
		(0.020)		(0.059)		(0.096)
RD		0.524 ***		0.117		0.089 ***
		(0.025)		(0.108)		(0.033)
ROE		0.023		0.020		0.027
		(0.061)		(0.031)		(0.040)
Size		0.355 ***		0.317 ***		0.324 ***
		(0.054)		(0.027)		(0.051)
Tobin		0.036 *		0.014		0.034 *
		(0.011)		(0.005)		(0.015)
Capital		0.368		0.300		0.331
		(0.037)		(0.035)		(0.037)
Ret		0.402 ***		0.433 ***		0.469 ***
		(0.060)		(0.035)		(0.058)
Constant	0.086 ***	0.182 ***	0.077 ***	0.563 ***	0.093 ***	0.183 **
	(0.011)	(0.271)	(0.062)	(0.056)	(0.083)	(1.200)
Firm-fixed effect	Control	Control	Control	Control	Control	Control
Year-fixed effect	Control	Control	Control	Control	Control	Control
Province-fixed effect	Control	Control	Control	Control	Control	Control
Observations	20,254	17,099	20,254	17,099	20,254	17,099
R-squared	0.689	0.692	0.695	0.658	0.791	0.728

Notes: The parentheses are the clustered standard errors. ***, **, and * indicate significant at the 1%, 5%, and 10% levels, respectively.

**Table 7:** Heterogeneity analysis of different pilot zones.

Variables	Guangdong	Jiangxi	Zhejiang	Guizhou	Xinjiang
	(1)	(2)	(3)	(4)	(5)
D	0.158 ***	0.139 *	0.263 **	0.116 ***	0.081 *
	(0.061)	(0.096)	(0.206)	(0.098)	(0.064)
Green × post	0.187 **	0.170 *	0.392 *	0.133 **	0.109 **
	(0.416)	(0.194)	(0.315)	(0.361)	(0.058)
Control variables	Yes	Yes	Yes	Yes	Yes
Constant	0.014	0.051 ***	0.055 ***	0.058 ***	0.012
	(0.027)	(0.016)	(0.015)	(0.015)	(0.022)
Firm-fixed effect	Control	Control	Control	Control	Control
Year-fixed effect	Control	Control	Control	Control	Control
Province-fixed effect	Control	Control	Control	Control	Control
Observations	2418	351	1599	247	403
R-squared	0.816	0.805	0.785	0.774	0.817

Notes: The parentheses are the clustered standard errors. ***, **, and * indicate significant at the 1%, 5%, and 10% levels, respectively.

**Table 8:** Heterogeneity analysis of listed company characteristics.

Variables	Ownership Attributes	Degree of Pollution	Scale of Listed Companies	Region Where Listed Companies Are Located
	(1)	(2)	(3)	(4)
D	0.020 ***	−0.104 **	0.026 *	0.344 **
	(0.104)	(0.114)	(0.120)	(0.242)
D× Owner	0.264 *			
	(0.154)			
D× Industry		0.390 ***		
		(0.144)		
D× Scale			0.189 *	
			(0.148)	
D× Region				0.309 **
				(0.248)
Control variables	Yes	Yes	Yes	Yes
Constant	1.498 ***	1.506 ***	1.593 ***	1.362 ***
	(0.182)	(0.109)	(0.095)	(0.079)
Firm-fixed effect	Control	Control	Control	Control
Year-fixed effect	Control	Control	Control	Control
Province-fixed effect	Control	Control	Control	Control
Observations	17,099	17,099	17,099	17,099
R-squared	0.539	0.595	0.586	0.549

Notes: The parentheses are the clustered standard errors. ***, **, and * indicate significant at the 1%, 5%, and 10% levels, respectively.

**Table 9:** Heterogeneity analysis of specific industry qualifications.

Variables	Services	Manufacturing	Energy	Processing	Engineering
	(1)	(2)	(3)	(4)	(5)
D	0.074	0.202 **	0.277 ***	0.186 *	0.094 **
	(0.063)	(0.133)	(0.141)	(0.165)	(0.135)
Control variables	Yes	Yes	Yes	Yes	Yes
Constant	2.427	3.603	3.214 *	3.516 **	4.696
	(5.431)	(2.787)	(1.754)	(1.683)	(3.296)
Firm-fixed effect	Control	Control	Control	Control	Control
Year-fixed effect	Control	Control	Control	Control	Control
Province-fixed effect	Control	Control	Control	Control	Control
Observations	3770	5863	3107	3510	4004
R-squared	0.848	0.867	0.834	0.820	0.859

Notes: The parentheses are the clustered standard errors. ***, **, and * indicate significant at the 1%, 5%, and 10% levels, respectively.

**Table 10:** Definitions and descriptive statistics of mediating variables.

Variable Statistics	Variable Definition	Observations	Means	Standard Deviation	Min	Max
Debt	Financial expenses/interest- bearing liability	17,757	0.0766	0.1715	−0.0378	0.8926
LDR	Long-term debt/total liability	17,099	0.0551	0.0859	−0.0063	0.4051

**Table 11:** Stepwise regression test for coefficients.

Variables	Model (7)	Model (8)		Model (9)	
	Grepatent	Debt	LDR	Grepatent	Grepatent
	(1)	(2)	(3)	(4)	(5)
D	0.073 **	−0.034 ***	0.093 **	0.072 *	0.145 *
	(0.090)	(0.009)	(0.010)	(0.090)	(0.098)
Debt				−0.225 ***	
				(0.441)	
LDR					0.104 **
					(0.510)
Control variables	Yes	Yes	Yes	Yes	Yes
Constant	1.490 ***	−0.035 ***	−0.011	1.473 ***	1.489 ***
	(0.183)	(0.012)	(0.010)	(0.108)	(0.182)
Firm-fixed effect	Control	Control	Control	Control	Control
Year-fixed effect	Control	Control	Control	Control	Control
Province-fixed effect	Control	Control	Control	Control	Control
Observations	17,099	17,099	17,099	17,099	17,099
R-squared	0.753	0.732	0.706	0.768	0.719

Notes: The parentheses are the clustered standard errors. ***, **, and * indicate significant at the 1%, 5%, and 10% levels, respectively.

## Data Availability

Not applicable.

## Supplementary Materials

The following supporting information can be downloaded at: https://www.mdpi.com/article/10.3390/ijerph191710882/s1, Supplementary Document, Table S1: Differences between green utility model patent applications and green invention patent applications; Table S2: The results of heterogeneity analysis with Greinva as the explained variable; Table S3: The results of heterogeneity analysis with Greuma as the explained variable; Table S4: Stepwise regression test for coefficients with Greinva as the explained variable; Table S5: Sobel test and bootstrap test with Greinva as the explained variable; Table S6: Stepwise regression test for coefficients with Greuma as the explained variable; Table S7: Sobel test and bootstrap test with Greuma as the explained variable.
