# Supplementary material for: Whether Green Finance Improves Green Innovation of Listed Companies—Evidence from China
Source: Int J Environ Res Public Health. 2022 Aug 31;19(17):10882. doi: 10.3390/ijerph191710882 (PMC9518455; doi:10.3390/ijerph191710882)
Supplement: Supplementary file 1 [file ijerph-19-10882-s001.zip › Supplementary document-GFRIPZ.pdf]

Whether Green Finance Improves Green Innovation Level of Listed Companies—Evidence from Green Finance Reform and Innovation Pilot Zone in China (Supplementary Document)

The supplementary document includes Table S1, Table S2, Table S3, Table S4, Table S5, Table S6 and Table S7, which are associated with the manuscript.

Green patent applications can be divided into two types: green invention patent applications and green utility model patent applications. The number of green patent applications of enterprises is the sum of the number of green invention patent applications and the number of green utility model patent applications. According to the provisions of the *Patent Law* (npc.gov.cn accessed on 15 May 2022), a utility model patent is a new technical scheme for practical use proposed for the shape, structure or their combination of the product. It is only related to the shape and structure of a product. While an invention patent refers to a new technical scheme proposed for products, techniques or their improvements, including product inventions and technique inventions. Product invention refers to the invention of objects created. Technique inventions refer to inventions made on processing techniques, manufacturing techniques, testing techniques, or product-used techniques. The differences between green utility model patent applications and green invention patent applications are presented in the following three aspects, presented in Table S1.

Table S1. Differences between green utility model patent applications and green invention patent applications are presented in supplementary materials.

Differentiated Aspects	Details
Creativity level	The creativity level of utility model patents is lower than that of invention patents. During the creative examination process of patents, an invention patent needs to have "prominent and substantive features and significant progress", while a utility model patent only requires "substantive features and progress".
Approval procedure	The *Patent Law* stipulates easier examination and approval procedure for utility model patent applications than for invention patents. Only preliminary examination is carried out for utility model patents. However, substantive examination is required in addition to preliminary examination for invention patents. Therefore, utility model patents generally can be certified about 6 months, while invention patents generally get certified about two years.
Application fees	The charges for utility model patent applications are lower than invention patent applications. In the case of no charge reduction, it costs 1,305 CNY to apply for a utility model patent until the patent certificate is obtained, whereas 4,605 CNY to apply for an invention patent.

To make our study comprehensive and rigorous, we present the results of heterogeneity analysis when Greinva and Greuma are selected as the explained variable respectively here, as shown in Table S2 and Table S3. Table S2 presents the results of heterogeneity analysis with Greinva as the explained variable, while Table S3 presents the results of heterogeneity analysis with Greuma as the explained variable. The conclusions are consistent with those drawn from Table 7 in the manuscript.

Table S2. The results of heterogeneity analysis with Greinva as the explained variable.

Variables	Ownership attributes (1)	Degree of pollution (2)	Scale of enterprises (3)	Region where enterprises locate (4)
D	0.020*	0.038**	0.073*	0.160***
	(0.062)	(0.063)	(0.059)	(0.154)
D× Owner	0.069**			
	(0.096)			
D× Industry		-0.180*		
		(0.091)		
D× Scale			0.224**	
			(0.088)	
D× Region				0.131*
				(0.158)
Control variables	Yes	Yes	Yes	Yes
Constant	0.269***	0.349***	0.225***	0.247***
	(0.053)	(0.101)	(0.051)	(0.055)
Firm-fixed effect	Control	Control	Control	Control
Year-fixed effect	Control	Control	Control	Control
Province-fixed effect	Control	Control	Control	Control
Observations	17099	17099	17099	17099
R-squared	0.575	0.577	0.617	0.626

Notes: The parentheses are the clustered standard errors. ***, ** and * indicate significant at the 1%, 5% and 10% levels, respectively.

Table S3. The results of heterogeneity analysis with Greuma as the explained variable.

Variables	Ownership attributes (1)	Degree of pollution (2)	Scale of enterprises (3)	Region where enterprises locate (4)
D	0.012*	0.114	0.040***	0.340**
	(0.102)	(0.112)	(0.118)	(0.241)
D× Owner	0.222**			
	(0.152)			

D× Industry		-0.396****		
		(0.142)		
D× Scale			0.202*	
			(0.146)	
D× Region				0.312
				(0.248)
Control variables	Yes	Yes	Yes	Yes
Constant	1.401***	1.398***	1.481***	1.573***
	(0.175)	(0.101)	(0.106)	(0.093)
Firm-fixed effect	Control	Control	Control	Control
Year-fixed effect	Control	Control	Control	Control
Province-fixed effect	Control	Control	Control	Control
Observations	17099	17099	17099	17099
R-squared	0.550	0.528	0.577	0.562

Notes: The parentheses are the clustered standard errors. ***, ** and * indicate significant at the 1%, 5% and 10% levels, respectively.

To make our study comprehensive and rigorous, here we present the results of mechanism analysis when Greinva and Greuma are selected as the explained variable respectively, as shown in Table S4, Table S5, Table S6 and Table S7. Table S4 presents stepwise regression test for coefficients with Greinva as the explained variable, and Table S5 presents sobel test and bootstrap test with Greinva as the explained variable. Table S6 presents stepwise regression test for coefficients with Greuma as the explained variable, while Table S7 presents sobel test and bootstrap test with Greuma as the explained variable. The findings of Table S4 and Table S6 are consistent with the conclusions drawn from Table 9 in the manuscript, and the findings of Table S5 and Table S7 are consistent with the conclusions drawn from Table 10 in the manuscript.

Table S4. Stepwise regression test for coefficients with Greinva as the explained variable.

**Variables**	**Model (7)**	**Model (8)**		**Model (9)**	
	**Greinva**	**Debt**	**LDR**	**Greinva**	**Greinva**
	**(1)**	**(2)**	**(3)**	**(4)**	**(5)**
D	0.045*	-0.075***	0.093***	0.046	0.087*
	(0.055)	(0.014)	(0.010)	(0.055)	(0.036)
Debt				-0.216**	
				(0.170)	
LDR					0.119*
					(0.233)
Control variables	Yes	Yes	Yes	Yes	Yes
Constant	0.347***	0.296***	0.247***	0.353***	0.227***

	(0.100)	(0.058)	(0.055)	(0.102)	(0.051)
Firm-fixed effect	Control	Control	Control	Control	Control
Year-fixed effect	Control	Control	Control	Control	Control
Province-fixed effect	Control	Control	Control	Control	Control
Observations	17099	17099	17099	17099	17099
R-squared	0.775	0.332	0.306	0.426	0.475

Notes: The parentheses are the clustered standard errors. ***, ** and * indicate significant at the 1%, 5% and 10% levels, respectively.

Table S5. Sobel test and bootstrap test with Greinva as the explained variable.

**Sobel test**	**Mediating variable：Debt**	**Mediating variable：LDR**
P-value	0.03164	0.01595
The proportion of mediation effect	59.5221%	23.3572%
Control variables	Control	Control
Observations	17099	17099
**Bootstrap test**	**Mediating variable：Debt**	**Mediating variable：LDR**
Confidence interval	[-0.1611, -0.1332]	[0.0143, 0.1629]
Control variables	Control	Control
Observations	17099	17099

Notes: Sobel test shows the P-value and the proportion of mediation effect. Bootstrap test shows confidence interval with 95%confidence level obtained by randomly sampling 1000 times estimation.

Table S6. Stepwise regression test for coefficients with Greuma as the explained variable.

**Variables**	**Model (7)**	**Model (8)**		**Model (9)**	
	**Greuma**	**Debt**	**LDR**	**Greuma**	**Greuma**
	**(1)**	**(2)**	**(3)**	**(4)**	**(5)**
D	0.066***	-0.034***	0.052***	0.491**	0.138*
	(0.088)	(0.009)	(0.014)	(0.081)	(0.095)
Debt				-0.299*	
				(0.431)	
LDR					0.069**
					(0.509)
Control variables	Yes	Yes	Yes	Yes	Yes
Constant	1.394***	0.499***	0.528***	1.374***	1.484***
	(0.176)	(0.105)	(0.093)	(0.096)	(0.106)
Firm-fixed effect	Control	Control	Control	Control	Control
Year-fixed effect	Control	Control	Control	Control	Control
Province-fixed effect	Control	Control	Control	Control	Control

Observations	17099	17099	17099	17099	17099
R-squared	0.749	0.432	0.506	0.693	0.588

Notes: The parentheses are the clustered standard errors. ***, ** and * indicate significant at the 1%, 5% and 10% levels, respectively.

Table S7. Sobel test and bootstrap test with Greuma as the explained variable.

**Sobel test**	**Mediating variable：Debt**	**Mediating variable：LDR**
P-value	0.04635	0.02188
The proportion of mediation effect	45.3885%	37.2466%
Control variables	Control	Control
Observations	17099	17099
**Bootstrap test**	**Mediating variable：Debt**	**Mediating variable：LDR**
Confidence interval	[-0.1758, -0.1593]	[0.0344, 0.1751]
Control variables	Control	Control
Observations	17099	17099

Notes: Sobel test shows the P-value and the proportion of mediation effect. Bootstrap test shows confidence interval with 95% confidence level obtained by randomly sampling 1000 times estimation.
